# Examining additive risk for cerebrovascular dysfunction in a novel late‐onset Alzheimer’s disease model

**DOI:** 10.1002/alz.092553

**Published:** 2025-01-03

**Authors:** Alaina M Reagan, Kevin P Kotredes, Rita O'Rourke, Sarah Herrick, Travis L Cossette, Michael Sasner, Gareth R Howell

**Affiliations:** ^1^ The Jackson Laboratory, Bar Harbor, ME USA; ^2^ University of Maine, Orono, ME USA; ^3^ Tufts University Graduate School of Biomedical Sciences, Boston, MA USA

## Abstract

**Background:**

Mechanisms driving cerebrovascular decline during Alzheimer’s disease and related dementias (ADRD) are poorly understood. Methylenetetrahydrofolate reductase (*MTHFR*) is an enzyme in the folate/methionine pathway. Variants in *MTHFR*, notably *677C>T*, are associated with ADRD. ∼30% of individuals carry at least one copy of *MTHFR^677C>T^*, causing a 50% decrease in MTHFR enzyme efficiency. Reduced efficiency can lead to elevated homocysteine in blood, resulting in vascular inflammation and increased risk for cerebrovascular damage. To examine vascular risk in a late‐onset Alzheimer’s disease (LOAD) model, MODEL‐AD created a mouse carrying known ADRD risk factors with the *Mthfr^677C>T^* variant. While plasma‐based protein biomarkers hold promise for identifying ADRD pathology, additional markers are necessary. Studies support eye exams as a synergistic strategy due to overlapping mechanisms of damage in retina and brain, so retinal phenotyping has been incorporated in our study.

**Methods:**

The *LOAD2.Mthfr^677C>T^* model was created by the IU/JAX/PITT MODEL‐AD Center by incorporating the *Mthfr^677C>T^* allele with the LOAD2 alleles (hAbeta/APOE4/Trem2*R47H). Cerebrovascular health is assessed in 4, 12, 18 and 24 month‐old mice by immunofluorescence to examine barrier integrity in cells that make up the cerebrovascular unit: endothelium, smooth muscle, pericytes, basement membrane, tight junctions, and extracellular deposition of blood products. Differential expression of vascular related genes/proteins is being examined by RNA‐sequencing and proteomics. *In vivo* retinal imaging, including optical coherence tomography and fluorescence angiography, and *ex vivo* retinal IF was performed to assess and compare neuronal and vascular health in the retina to the brain.

**Results:**

All mice have been aged, and in vivo assays completed. Compared to LOAD2 controls, LOAD2*.Mthfr^677C>T^* mice do not show significant changes in frailty, activity, or cognition. At 24 mos, transcriptomic and proteomic data suggest LOAD2 and LOAD2.*Mthfr^677C>T^* show some expression changes similar to those seen in human AD. By immunofluorescence, like LOAD2, *Mthfr^677C>T^* mice do not develop ThioS‐positive amyloid plaques, but assessment of vascular‐specific changes is in progress. Plasma and retinal biomarker analysis is also in progress.

**Conclusions:**

MODEL‐AD continues to create strains that develop endophenotypes of LOAD for preclinical testing. Ultimately, this work will aid in the identification of novel therapeutic approaches that reduce cerebrovascular pathology in ADRDs.

